# Early Exposure to Soy Isoflavones and Effects on Reproductive Health: A Review of Human and Animal Studies

**DOI:** 10.3390/nu2111156

**Published:** 2010-11-23

**Authors:** Elsa C. Dinsdale, Wendy E. Ward

**Affiliations:** Department of Nutritional Science, Faculty of Medicine, University of Toronto, Toronto, Ontario, Canada; Email: elsa.dinsdale@utoronto.ca

**Keywords:** isoflavones, soy, reproductive health, infants, rodent models

## Abstract

Soy isoflavones are phytoestrogens with potential hormonal activity due to their similar chemical structure to 17-β-estradiol. The increasing availability of soy isoflavones throughout the food supply and through use of supplements has prompted extensive research on biological benefits to humans in chronic disease prevention and health maintenance. While much of this research has focused on adult populations, infants fed soy protein based infant formulas are exposed to substantial levels of soy isoflavones, even when compared to adult populations that consume a higher quantity of soy-based foods. Infant exposure, through soy formula, primarily occurs from birth to one year of life, a stage of development that is particularly sensitive to dietary and environmental compounds. This has led investigators to study the potential hormonal effects of soy isoflavones on later reproductive health outcomes. Such studies have included minimal human data with the large majority of studies using animal models. This review discusses key aspects of the current human and animal studies and identifies critical areas to be investigated as there is no clear consensus in this research field.

## 1. Introduction

Soy protein based infant formula (SBIF) has been used throughout the world for over 100 years [1]. SBIFs were initially developed as an alternative to cow’s milk based formula for infants with immunoglobulin E-mediated milk allergies, post-infectious diarrhea due to lactose intolerance, galactosemia, or for infants who required a vegan substitute [1]. SBIFs were originally prepared from soy flour which had lower digestibility and lower protein content compared to the soy protein isolate (SPI) which is used currently [2]. SBIF have been further modified to include methionine, iodine, carnitine, taurine, choline and inositol [1]. According to the Infant Formula Act of 1980, amended in 1986, SBIFs meet all nutritional requirements for term infants [3]. Data from North America suggest that approximately 37.2% to 43.8% of infants are formula fed three to six months postpartum [4]. Recent data suggests the prevalence of feeding SBIF is 20–25% in Canada [5] and the United States [6] and markedly lower (2–3%) in the United Kingdom [7] and Australia [8].

### 1.1. Isoflavones in Soy Protein Based Infant Formulas (SBIF)

SBIFs represent a significant source of soy isoflavones with potential hormone-like activities [9] as they contain a diphenolic ring that allows them to bind to the estrogen receptor (ER) [10]. There are two predominant types of soy isoflavones, daidzein and genistein, which preferentially bind to ER‑β [11]. In addition to exerting hormone-like effects, isoflavones may also act through a non‑hormonal mechanism by inhibiting tyrosine kinases and inducing some growth arrest and apoptosis [12].

Although soy isoflavones are weakly estrogenic, approximately 10^2^ to 10^3^ fold less potent than endogenous estrogen [13], infants consuming SBIF have extremely high levels of serum isoflavones [14]. One study reported the isoflavone content of the major brands of commercially available SBIFs as well as the serum concentrations of isoflavones (including genistein, daidzein and its metabolite, equol) in four month old infants exclusively fed SBIF, cow’s milk formula, or human breast milk. The levels of isoflavones in five different SBIFs varied from 32 to 47 mg isoflavones/L of formula [9,14]. Thus, infants fed these formulas are exposed to 5.7–11.9 mg isoflavones/kg body weight during the first four months of life. Compared to adults consuming a soy rich diet, which could contain approximately 0.71 mg/kg body weight (assuming a body weight of 70 kg) [15], infants fed SBIF are exposed to a 6–11 fold higher level of isoflavones on a body weight basis than adults. Additionally, circulating isoflavone levels of these infants were 13,000–22,000 times greater than circulating levels of 17-β-estradiol [9].

### 1.2. Studying the Effects of Soy Isoflavones in SBIF

Currently, there is debate regarding the physiological impact of soy isoflavone consumption and whether or not it warrants concern for infants fed SBIF. Because only one study has reported on reproductive health outcomes at adulthood [16], it is not possible to know whether feeding infants SBIF is associated with negative effects on reproductive health. Thus, due to the paucity of human data, it may be useful to assess findings from studies using animal models to more fully understand effects in humans. Unquestionably there are considerations that need to be made when extrapolating findings from animal models to humans (Table 1). Human and animal studies have different challenges and we propose that both types of studies are important to achieve a comprehensive understanding of the biological effects of soy isoflavones on reproductive health. 

**Table 1:** The challenges of designing and conducting studies in humans or using rodent models to study the effects of soy isoflavones on reproductive health.

Humans Studies	Rodent Models
Long-term time commitment to follow birth cohort through to adulthood; Extensive and long-term continuous funding required; Consideration of environmental factors that can impact reproductive health (***i.e.***, exposure to endocrine disruptors, physical activity, smoking, age, education, diet and disease history); Limited to measurement of noninvasive outcomes (***i.e.***, organ size, menstrual cycle, fertility) that may provide limited insight into mechanisms of action.	Species-related differences in digestion, absorption and metabolism of isoflavones; Administering purified isoflavone or isoflavones as part of SBIF; Route of isoflavone administration: Oral feeding versus subcutaneous injection; Frequency of isoflavone administration: Once daily dose or multiple doses per day; Composition of soy isoflavone mixture to mimic the ratio and combination of isoflavones present in SBIF; Equating the timing of the life cycle between rodents and humans.

#### 1.2.1. Challenges of Human Studies

Some of the major challenges of designing studies and collecting human data regarding the effects of soy isoflavones on reproductive health are outlined in Table 1.

##### 1.2.1.1. Long-Term Time Commitment

Determining the effect of SBIF on human reproductive development requires a long-term commitment, both on the part of the investigator(s) as well as the subjects. Ideally, children would be monitored before puberty and until sexual maturity is reached. This would allow potential differences in reproductive organ development to be discerned. Furthermore, reproductive capacity should be assessed throughout potential child-bearing years. As with all long-term studies, subject compliance and retention over this long time period will be difficult. Retrospective cohort studies of individuals who consumed SBIF are another strategy and indeed one such study has been reported [16]. A limitation of retrospective studies is recall bias. Unless a birth cohort with detailed records of infant feeding from birth through at least the first months of life exist, it is difficult to accurately determine to the type of feeds an infant received. It is also useful to collect data at various life stages in order to determine if both reproductive development and capacity are normal. Retrospective cohort studies are still very time intensive and recruiting sufficient number of participants and controlling for the multitude of factors that may affect reproductive capacity may be particularly challenging.

##### 1.2.1.2. Expensive and Long-Term Funding Required

From a practical standpoint, prospective studies in particular, as discussed above, are costly and require long-term funding. Retrospective studies would also require a substantial level of funding. 

##### 1.2.1.3. Environmental Factors

Accounting and controlling for various environmental differences that subjects either would be, or were exposed to, is difficult to control. Other factors such as level of physical activity, dietary patterns, smoking and history of disease may be possible to correct for but it is considerably more difficult to determine the level of subject exposure to known environmental endocrine disruptors. Examples of such estrogenic compounds include chemicals used in cosmetics and persistent organic pollutants [17]. 

##### 1.2.1.4. Limited to Measurement of Noninvasive Outcomes

Undisputed is the fact that data from human subjects is ideal to determine if isoflavones in SBIF modulates reproductive development. However, human studies are limited to measurement of fairly noninvasive outcomes such as pubertal maturation, sexual orientation, body weight, menstrual characteristics, congenital characteristics of subject offspring and hormone dependent cancers (*i.e.*, testicular, ovarian, breast, prostate). Indeed, the one retrospective study to date included many of these measurements [16]. Measurement of serum hormone levels, reproductive organ size and morphology would provide a more comprehensive understanding of biological effects of SBIF in human infants. 

#### 1.2.2. Challenges of Using Rodent Models

Some of the major challenges of designing studies and collecting rodent data to determine the effects of soy isoflavones on reproductive health are outlined in Table 1.

##### 1.2.2.1. Species Related Differences

Rats, mice, marmosets and piglets have been used in animal studies and it is known that there are some species related differences in absorption and metabolism of soy isoflavones [18]. There are, however, some aspects important similarities in isoflavone metabolism among species. For example mouse pups, like human infants, do not produce equol which is the more estrogenic metabolite of daidzein [14]. 

##### 1.2.2.2. SBIF *versus* Purified Soy Isoflavones

Isolated soy isoflavones are frequently used in animal studies but it is unknown whether they act differently when present in SBIF, a complex mixture of phytochemicals and peptides. Using rodent models it is not possible to deliver sufficient levels of isoflavones by using SBIF as the volume required is too high. This is likely the basis for why most investigators have administered purified isoflavones rather than SBIF for rodents. A few studies have fed soy formula directly to animals, such as marmosets or pigs and thus when reviewing the animal data it is important to know the form of isoflavones provided. 

##### 1.2.2.3. Route of Administration—Oral *versus* Subcutaneous Injection

Additional considerations include the route of isoflavone administration in a study. There have been differences reported in the serum isoflavone levels in rodents depending on whether the isoflavone mixture was given orally or delivered by subcutaneous injection [19]. We have previously shown that subcutaneous injection of genistein and daidzein in mice results in serum isoflavone levels that are comparable to human infants fed SBIF [20]. This is significant because it suggests that the use of subcutaneous injection is useful even though injected genistein bypasses first pass metabolism in the gut. Oral feeding in rodent neonates is possible but due to the small size of the pup it is difficult to ensure that all of the isoflavone mixture is consumed during an oral feeding (measurement of serum levels are needed to determine the level of isoflavone exposure). Another issue with oral feeding from birth through the first days of life includes the risk of aspiration requiring premature euthanization. One study used a combination of subcutaneous injection and oral feeding and examined the effects of genistein on postnatal development from birth until PND 21 in rat pups. The authors state that it was not technically practical to orally gavage the rat pups from birth and thus subcutaneous injections were used from birth until PND 7 [21] and pups were fed orally thereafter. Oral feeding may be more desirable to more closely mimic the human infant scenario, and whether differences in metabolism result in different biological effects requires further study.

In one study, mice were given varying doses of genistein in order to determine the oral dose that would achieve serum levels close to human infants consuming SBIF. It was determined that 5–20 mg oral genistein/kg body weight did not have a measurable effect on serum genistein levels but an oral dose of 50 mg genistein/kg body weight resulted in serum levels of 2–3 µM [22] and others have shown that subcutaneous injection at this same dose (50 mg/kg body weight) also results in similar serum levels (1–5 μM) [23]. This is similar to the 1–5 µM total serum isoflavone levels observed in human infants [14]. This demonstrates that there may be no difference in serum levels after oral or subcutaneous administration and that the dose of 50 mg isoflavones/kg body weight may achieve serum isoflavone levels close to human infants [22,23]. However, whether different routes of administration (*i.e.*, oral or subcutaneous injection) result in similar or dissimilar serum isoflavone levels requires direct comparison within a study. Another study that administered markedly lower doses of isoflavones (5 mg genistein/kg body weight and 2 mg daidzein/kg body weight by subcutaneous injection) resulted in serum levels of 2.61 ± 0.97 μM and 1.07 ± 0.19 μM respectively [20]. Such findings demonstrate the need for further study in order to determine appropriate oral and subcutaneous doses to mimic physiological relevant serum isoflavone levels. 

##### 1.2.2.4. Frequency of Exposure

Notably, studies that have successfully used oral feeding have administered the mixture once per day. In order to truly represent the human infant experience, multiple oral feedings would be required. This aspect requires future study.

##### 1.2.2.5. Composition of Isoflavones

Genistein, which is considered the most abundant soy isoflavone found in SBIF [14] has been the focus of investigation and is often administered in isolation. Genistein, however, is not the only active isoflavone in SBIF. Daidzein is also present in soy formula and accounts for approximately 28.7% of total isoflavone content (genistein accounts for approximately 67.1% of total isoflavones) [14]. Therefore, the ratio and dose of isoflavones should be comparable to SBIF, and both genistein and daidzein should be administered if isolated soy isoflavones are used rather than SBIF.

##### 1.2.2.6. Equating the Timing of the Life Cycle of Rodents with that of Humans

The timing of when isoflavone exposure should take place using an animal model, in order to mimic the first year of life in humans, is debatable. Mice suckle for the first 21 days of life and thus it makes sense that soy isoflavone exposure should take place during the age of suckling to mimic the stage of development in which human infants may be fed SBIF. A difficulty is that mice also start to reach sexual maturation shortly thereafter, being able to breed by six weeks of age, whereas humans have a much longer duration before sexual maturation takes place. 

### 1.3. Safety of Soy Isoflavones for Reproductive Health

Several countries have controlled the availability of SBIF because of concern regarding safety of isoflavone exposure in early life and reproductive development. In Europe, for instance, SBIF is only available by prescription [24]. In 1996, the United Kingdom Committee on Toxicity of Chemicals in Foods, Consumer Products and the Environment considered the presence of phytoestrogens, such as soy isoflavones, in SBIF and subsequently supported the advice of the U.K. Department of Health, that human breast milk and cow’s milk are preferred sources of nutrition for infants and SBIF should only be recommended when there is clinical indications [25]. The Working Group identified the need for further studies, and stated that due to a paucity of data it is not possible to make a final conclusion. The Canadian Paediatric Society, Dietitians of Canada, Health Canada and the American Academy of Pediatrics as well as many other organizations recognize that breastfeeding is the optimal method for feeding infants [9,10]. The use of SBIFs is recommended for only those infants who cannot have dairy-based products because of health, cultural or religious reasons. Nonetheless, significant numbers of infants are fed SBIFs during the first year of life.

In the United States, The National Toxicology Program Center for the Evaluation of Risks to Human Health Reproduction (NTP-CERHR) convened in December of 2009 to evaluate the safety of SBIF. A panel of experts reviewed and evaluated the quality and strength of available scientific data regarding early exposure to SBIF or its isoflavones and how it may impact human development [26]. In 2006, the NTP previously convened to evaluate soy formula and genistein, but did not complete the evaluation or issue a final statement due to insufficient data [27]. The substantial number of studies that were released from 2006–2009 prompted the NTP-CERHR to revisit this topic. The NTP-CERHR and expert panel has since concluded that there is “minimal” concern due to a paucity of data focusing on early, critical stages of the life cycle [26]. Moreover, it was stated that the design of some studies are not ideal for evaluating the safety of SBIF. These aspects have been summarized and discussed in secton 1.2 and Table 1. However, the relatively high number of studies in experimental animals, and a few studies in humans, reported some effect of isoflavones on reproductive health, and this raised the level of concern from “negligible” to “minimal”. The report also stated that there is insufficient human data to form a definitive conclusion [26].

The objective of this review is to examine the current scientific data regarding soy isoflavones and effects on reproductive health. Since there are few studies examining the effects of SBIF in humans, it is necessary to include animal studies, mainly in rodents, to discern the effect of soy isoflavones on human health but keeping in mind the limitations/challenges in extrapolating findings from animal studies to human infants. Before discussing the findings from these studies it is useful to review the indicators of reproductive health that have been measured and what information they provide regarding effects on reproductive health (Table 2). These indicators are grouped according to whether they are indicators of sexual maturation or endocrine disruption. Multiple measures should be used when determining the effect of an estrogenic compound on reproductive development. 

**Table 2:** Indicators of sexual maturation and endocrine disruption in rodent models.

Sexual Maturation	
**Preputial Separation**	The separation of the foreskin of the penis from the glans, preputial separation (PPS) is an early marker of the progression of puberty.
**Vaginal Opening**	The initial marker of the rise in circulating estrogen that signifies the onset of puberty and first ovulation followed by the start of estrous cycling.
**Endocrine Disruption**	
**Anogenital Distance (AGD)**	The distance between the anus and genital protuberance in newborns of various species including mouse and rat is used as the sole external sex-differentiating marker (longer in males compared to females) and is used to determine whether or not endocrine disruption has occurred. Under-masculinization is said to have occurred if AGD is shortened compared to control animals.
**Sex Organ Histology**	Changes in morphology of the mammary gland, ovary, uterus, testes are indicators of estrogenic effects that may ultimately be manifested as enhanced or reduced fertility.
**Sex Organ Weight**	Higher weight of uterus, ovaries, testes, or prostate may indicate estrogenic effects due to higher rates of cell proliferation within the organ.
**Serum Hormones**	Measurement of sex steroid hormones (***i.e.***, LH, FSH, GnRH, estradiol, progesterone, testosterone) demonstrates estrogenic perturbations in the endocrine system.
**Estrogen Receptor Activity**	Elevated transcription of ER-ß or ER-α is indicative of higher estrogenic activity.
**Estrous Cycle**	Length of time spent in each phase of estrous cycle can be used to understand if fertility may be altered, ***i.e.***, if an animal is in prolonged diestrus, lower fertility may result.
**Lordosis Quotient**	A measure of sexual behavior and is calculated by dividing the number of lordoses (inward curving of a portion of the vertebral column) by the number of mounts.

## 2. Results and Discussion

Review of studies to date show that interventions with isoflavones have been conducted at different life-stages, and in some studies the intervention occurred over more than one life-stage. This review focuses on studies in which animals were directly exposed to soy isoflavones during suckling since the objective was to review studies that relate to infants fed SBIF. The doses of soy isoflavones and the route of administration used in the studies reviewed are summarized in Table 3. Of note is that few studies measured serum levels of soy isoflavones. Without knowing serum levels of isoflavones, it is difficult to directly compare findings to human infants.

**Table 3:** Summary of isoflavone doses, route of administration and serum measurements in rodent models studying reproductive health.

	Dose	Route of delivery	Serum Isoflavone Levels	Ref.
**Females**	0.0001–100 mg genistein or daidzein/kg body weight	SC	NM *	[28]
	0.5, 5, 50 mg genstein/kg body weight	SC	NM *	[29],[30],[31]
	12.5, 25, 50 or 100 mg genistein/kg body weight	Oral	NM *	[32]
	50 mg genistein/kg body weight	SC	NM *	[33],[34],[35]
		Oral		
	5, 20, 50, 100 mg genistein/kg body weight	Oral	5, 20 and 100 mg genistein/kg body weight: below desired range;	[22]
			50 mg genistein/kg body weight resulted in desired serum range of: 2–3 µM	
	Oral genistin: 6.25, 12.5, 25 or 37.5 mg/kg body weight/day; Oral genistein: 25, 37.5, 75 mg/kg/day	Oral	Serum levels of oral GIN and GEN were measured at 37.5 mg/kg body weight; GEN AUC/dose = 2.4; GIN AUC/dose = 0.34	[19]
	Subcutaneous genistein: 12.5, 20, 25 mg/kg body weight	SC	NM *	[19]
	0.2, 2, 4, 40 mg genistein/kg body weight (sexes combined)	SC	SC 4 mg genistein/kg body weight: 0.99 µg/equivalents/h/mL; 40 mg/kg body weight: 5.82 µg/equivalents/h/mL	[21]
		Oral	40 mg genistein/kg body weight: 0.53 µg/equivalents/h/mL	[21]
	83 mg genistein or daidzein/kg body weight	SC	NM	[36]
	500 mg genistein/kg body weight	SC	NM	[37]
**Males**	4 mg genistein/kg body weight	SC	NM	[38]
	1.6–3.5 mg isoflavones/kg body weight	Oral	NM	[39],[40]
	0.2, 2, 4, 40 mg genistein/kg body weight (sexes combined)	SC	4 mg genistein/kg body weight: 0.634 µg/equivalents/h/mL; 40 mg/kg body weight: 5.82 µg/equivalents/h/mL	[21]
		Oral	40 mg genistein/kg body weight:	[21]
			0.53 µg/equivalents/h/mL	
	12.5 25, 50 or 100 mg genistein/kg body weight	Oral	NM	[32]

* Previously measured serum genistein levels of 1–5 μM after subcutaneous injection of 50 mg genistein/kg body weight are reported [23]; Human infants are exposed to 5.7–11.9 mg soy isoflavones/kg body weight resulting in serum concentrations of 1–5 µM total isoflavones [14].

### 2.1. Male and Female Reproductive Health: Human Studies (Table 4)

To date, few studies have investigated the impact of early life consumption of SBIF on reproductive function in adult life (Table 4). Strom *et al.* reported no differences in more than 30 reproductive health outcomes including the age of onset of puberty and reproductive function in males. Prolonged menstruation as well as increased discomfort during menstruation was more frequently reported in the female group [16]. Increased vaginal cell maturation has been reported in female infants at six months of age, and was considered to be an estrogenic effect attributed to the consumption of SBIF in early life [41]. Other outcomes, including vaginal discharge and breast and genital development were not altered [41]. Another study, which focused on infants at two years of life did however demonstrate differences in breast development [42]. For example, breast tissue was more prevalent in infants fed SBIF at two years of life compared to those fed cow’s milk-based formula or breast milk [42]. Currently, The Beginnings Study, a longitudinal prospective study, is in progress at the Arkansas Children’s Nutrition Center to compare growth, development, and health of breastfed or formula-fed children [43]. Findings to date have shown that formula-feeding itself, without discriminating between type of formula, results in greater ovarian volume, increased numbers of ovarian cysts per ovary and lower testicular volume [43]. Consideration in the interpretation of these findings is that 32% of infants in the SBIF group did not consume SBIF until 4–8 weeks of age. Because timing of exposure may modulate effects of later health it will be important to further investigate how timing of exposure may influence reproductive outcomes at later stages of development (*i.e.*, beyond four months of age).

### 2.2. Female Reproductive Health: Animal Studies (Table 5)

Studies that investigated the long-term consequences of soy isoflavone exposure during suckling have demonstrated differing effects at adulthood (Table 5).

**Table 4:** Studies examining the effect of soy isoflavone exposure in early life on human development.

Objective	Sample Size	Age of Subjects	Intervention Duration	Reproductive Health Outcomes	Findings
Retrospective cohort study to determine the association between soy infant formula consumption and health in adulthood with focus on reproductive health; Self-reported pubertal maturation, menstrual and reproductive history, height and usual weight [16] .	***n*** = 248 SBIF ***n*** = 563 cow’s milk formula	Adults aged 20–34	Adults as infants were treated from age 9 days or before to16 weeks of age; Cow’s milk formula; SBIF (soy isoflavone content of the formula was unknown)	**Women:** adult height, weight body mass index, pubertal maturation number of days between periods number of days requiring pads or tampons, regularity of menstrual period, menstrual flow, pain with menstrual period, physical symptoms of pain, breast tenderness during menstrual cycle, premenstrual symptoms, breast size, reproductive outcomes, and education level attained as a proxy measure for intelligence; **Men:** adult height, usual weight, and education level, pubertal maturation and pregnancy outcomes in sexual partners impregnated by the male study subjects, congenitalmalformations in the offspring of study subjects, hormonal disorders, testicular cancer in men, and homosexual orientation.	**Men and Women:** No statistically significant differences were reported between groups in either men or women for more than 30 outcomes; **Women:** Significantly longer menstrual bleeding and greater discomfort during menstruation.
To pilot techniques for assessing infants’ responses to the withdrawal from maternal estrogen and gathered data on breast and genital development in infants at different ages in infants who have consumed SBIF, cow’s milk formula or exclusively breast milk [41]	***n*** = 72 equally distributed in SBIF, cow’s milk formula and exclusive breast fed	37–41 weeks of age	37–41 weeks of age until 6 months of age	Breast adipose tissue; Breast bud and testicular volume; Observed breast and genital development; Vaginal wall cytology; Vaginal discharge	Breast tissue was maximal at birth and disappeared in older children, consistent with waning maternal estrogen; Genital development did not change by age; Vaginal wall cells showed maximal estrogen effect at birth and then reverted as normal; Female infants on SBIF appeared to show reestrogenization at 6 months, by increased maturation in vaginal cells
To evaluate the estrogenic effect of soy-based formulas in female infants [42]	***n*** = 50–92 SBIF for more than 3 months ***n*** = 602–232 Milk group (both breast milk and cow’s milk)	3–24 months of age	3–24 months of age	Breast development	No differences in breast bud prevalence during the first year of life; Infants fed SBIF did not demonstrate a decline in the prevalence of breast tissue during the second year of life, unlike other groups
To determine if differences exist in hormone-sensitive organ size between infants who were fed soy formula (SBIF), milk formula (MF), or breast milk (BM) [43]	***n*** = 40 BM ***n*** = 41 MF ***n*** = 39 SBIF	Age 4 months; SBIF exclusively fed from birth up to 8 weeks of age and continuing until 4 months of age (32% did not switch to SF until 4–8 weeks of age); MF from birth to 4 weeks until 4 months of age; BM from birth until 4 months of age	BM, MF or SBIF for 4 months	Anthropometry; Body composition; Breast buds, uterus, ovary, prostate and testicular volume	In both formula groups males had lower testicular volume, and females had greater ovarian volume, increased numbers of ovarian cysts per ovary; Other measures were not significantly different between the control and SBIF groups

**Table 5:** Studies in female animal models examining the effects of soy isoflavone exposure during early life.

Objective	Sample Size	Subjects	Intervention: Route of administration and dosage	Duration of Intervention	Reproductive Health Outcomes	Findings
		(age at time of intervention)				
To determine if the orally administered genistin (GIN), the glycosylated form of genistein (GEN), causes adverse effects on the developing reproductive tract GIN is most predominant in soy isoflavone formulas, but infants consuming SBIF have high circulating levels of GEN [19]	***n*** = 4–16 mice/group	CD-1 mice, PND 1	SC: genistein: 12.5, 20, 25 mg/kg body weight Oral genistin (GIN):6.25, 12.5, 25 or 37.5 mg/kg body weight Oral genistein (GEN): 25, 37.5, 75 mg/kg/day	PND 1–5	**SC GEN, Oral GEN, Oral GIN** Uterine wet weight gain Induction of estrogen-responsive gene, lactoferrin (LF) **GIN Group only ** Vaginal opening Estrous cycling Fertility Morphologic alterations in ovary/reproductive tract	**SC GEN, Oral GEN, Oral GIN** 20–33% more oral GIN was needed to elicit uterine wet weight gain compared to SC GEN but similar response was observed Oral GEN uterine wet weight gain only observed at much higher doses of 75 mg genistein/kg body weight Induction of LF gene **Oral GIN: ** Increased incidence of multioocyte follicles in the ovaries Delayed vaginal openingAltered estrous cycling Decreased fertility Delayed parturition
To develop a mouse model that more closely mimics the oral genistein exposure and total serum genistein concentrations. To assess reproductive and nonreproductive organs after dosing and during development [22]	Not determined	C57BL/6 mice, PND 1	Oral genistein-soy formula emulsion: 5, 20, 50, 100 mg/kg body weight	PND 1–5	Serum genistein concentration Thymic and uterine weights Follicle numbers Immunohistochemistry for progesterone receptor	**5, 20, 100 mg genistein/kg body weight: below desired range of serum genistein** **50 mg genistein/kg body weight** Increased uterine weight Downregulation of progesterone receptor in uterine epithelia Increased incidence of multioocyte follicles Decrease in thymic weight Altered estrous cyclingNormal fertility
To determine the effects of oral exposure to genistein in order to assess human risk following oral ingestion of genistein [21]	Not determined	Alderley Park rat PND 1	**PND 1–6** SC Genistein: 0.2 or 2 mg/kg body weight **PND 7–21** Oral gavage Genistein: 4 or 40 mg/kg body weight Control: corn oil	PND 1–21	Serum LH, FSH, estradiol, progesterone Vaginal opening Estrous cycling Sex organ weights GnRH	**40 mg genistein/kg body weight:** Increased uterus weights at PND 22 Advanced mean day of vaginal opening Induced permanent estrus Decreased progesterone in mature females **4 mg genistein/kg body weight:** No effects
To measure the estrogenic responses of several phytoestrogens including genistein, daidzein and compare them over a dose range and measuring the transcriptional activation of the estrogen receptor (ER) and an ***in vivo*** immature mouse uterotrophic assay [28]	Not determined	CD-1 mice, PND 17	SC Genistein and daidzein doses 0.00001 to 1000 mg/kg body weight Positive controls: Diethylstilbestrol (DES)17β-estradiol: 0.01 to 1,000,000 µg/kg body weight Negative control: corn oil	3 consecutive days (PND 17, 18,19)	Uterine wet weight Uterine epithelial height Uterine gland number	**Daidzein treatment: ** Did not demonstrate any increase in uterine epithelial cell height; Increase in uterine gland number; Did not demonstrate an increase in uterine wet weight; **Genistein treatment: ** Increase in uterine wet weight; Increase in uterine epithelial cell height; Increase in uterine gland number
To determine the biochemical effect of genistein as the induction of ectopic expression of ER in granulosa cells, a morphological effect as the induction of multioocyte follicles (MOFs) in the ovary, and a functional effect as the altered ovarian response to superovulation treatment [29]	***n*** = 16/group	CD-1 mice, PND 1	SC Genistein: 1, 10, 100 µg/pup/day (approximately 0.5, 5 or 50 mg/kg body weight)	5 days PND 1–5	ER-ß and ER-α expression and distribution in ovarian tissues The impact of genistein on ER expression, ovulation and the development of multioocyte follicles	ER-β transcript expression predominated in the ovaries in all stages of life and over ER-α and increased with age Genistein did not change ER-β expression but ER-α expression increased on days 5 and 12 ER-β was immunolocalized to granulosa cells ER-α was immunolocalized in interstitial and thecal cells Genistein caused major increase in ER-α expression in granulosa cells Superovulated mice had an increase in the number of ovulated oocytes at the lowest dose Dose-related increase in multioocyte follicles (MOFs)
To determine the the processes involved in altered mammary gland growth and development after neonatal genistein treatment [30]	***n*** = 3–8/group	CD-1 mice, PND 1	SC Genistein 0.5, 5 or 50 mg/kg body weight	PND 1–5	Development of the mammary gland	**4-week:** No morphological differences were observed in development **5-week:** Gen50 group had stunted development(less branching ) decreased numbers of terminal end buds **6-week:** Gen50 had decreased number of terminal end buds, Gen 0.5 treated mice had advanced development with increased ductal elongationIncreased levels of progesterone receptor protein and estrogen receptor-β mRNA in Gen0.5-treated mice compared with controls ER-α expression decreased after all doses of Gen treatment Gen50 treated mice were unable to deliver live pups
To study the effects of neonatal genistein exposure on attainment of puberty and fertility [31]	Not determined	CD-1 mice 2, 4, 6 months of age	SC Genistein:0.5, 5 or 50 mg/kg body weight	PND 1–5	Vaginal opening Fertility Implantation and pregnancy Ovarian function (number of corpus luteum and ovarian capacity) Estrous cyclicity Serum hormone levels (estradiol and progesterone) before puberty	Genistein treated mice had prolonged estrous cycles that had a dose and age-related increase Pregnancy loss was attributed to fewer implantation sites and increased resorption Low dose genistein treated mice had increased numbers of corpora lutea compared to controls High dose genistein treated mice had fewer corpora lutea Similar levels of serum estrogen, progesterone and testosterone were observed before and during pregnancy Mice treated with Gen-50 did not deliver live pups
To evaluate whether early exposure of neonates to genistein has any effect on the development of sexual organs and/or reproductive performance [32]	***n*** = 10–24/group	Sprague-Dawley rats PND 1	Oral gavage Genistein:12.5, 25, 50 or 100 mg/kg body weight Control: corn oil	PND 1–5	Fertility Vaginal Opening Estrous cycling Histopathological changes in the reproductive organs	Fertility was disrupted at 100 mg genistein/kg body weight Age at vaginal opening was not altered Estrous cycle: genistein-treated had cycle had variation in the amount of time spent in each phase and this was not dose responsive, cycle length was normal Histopathological changes in the uterus and ovary at 100 mg genistein/kg body weight
To study the formation of multioocyte follicles (MOFs) and potential disruption of the development of the ovary by genistein on ovarian differentiation [33]	***n*** = 24–48/group	CD-1 mice, PND 1	SC Genistein 50 mg/kg body weight (~100 μg/pup/day)	PND 1–5	Ovarian differentiation	**Genistein treatment:** Fewer single oocytes Higher percentage of oocytes not enclosed in single follicles Oocytes nest breakdown was prolonged Fewer oocytes undergoing apoptosis on neonatal day 3
To determine the long-term carcinogenic potential in mice treated neonatally with genistein or DES with equal estrogenic dose [34]	***n*** = minimum 8/group	CD-1 mice, PND 1	SC Genistein: 50 mg/kg body weight DES:0.001 mg/kg body weight Negative control: corn oil	5 days PND 1–5	Incidence of uterine adenocarcinoma Uterine weightCorpora lutea absence Abnormalities in the oviductOvarian tumor	Higher incidence of uterine adenocarcinoma at 18 months with genistein and DES; Higher uterine weight gain with genistein and DES; Higher absence of corpora lutea with genistein and DES
To elucidate the mechanism by which gensitein leads to infertility [35]	Not determined	CD-1 mice, PND 1	SC Genistein 50 mg/kg body weight Control: corn oil	PND 1–5	Oocyte developmental competence Timing of embryo loss	**Genistein treatment:** Females were not capable of supporting normal implantation of control embryos Oocytes were competent but the oviductal environment and the uterus have abnormalities that result in reproductive failure Complete infertility observed
To examine the effect of phytoestrogens on female sexual behavior and ovarian cyclicity [36]	***n*** = 9–10/group	Wistar rats PND 1	SC Genistein 1 mg/day Daidzein 1 mg/day Control: sesame oil	PND 1–5	Estrous cycle Vaginal Opening Ovary histology Lordosis quotient (feminine sexual reflexes)	**Genistein treatment: ** Prolonged estrous cycle Smaller ovaries and no corpora lutea compared to control or DZ group Low lordosis quoteint **Daidzein treatment: ** Corpora lutea seen but ovaries were smaller compared to controls High lordosis quotient
	
	
To investigate the potential of genistein to protect against the development of breast cancer and to cause reproductive and developmental toxicity [37]	Not determined	Prepubertal female, suckling, Sprague-Dawley rats	SC Genistein 500 mg/kg body weight Oral gavage Carcinogen: Dimethylbenz[a]anthracene (DMBA) 80 mg/kg body weight	Genistein:3 days, every second dayPND 16, 18, 20 DMBA: PND 50	Mammary gland differentiation and cell proliferation in the presence of carcinogen DMBA; Offspring body weights; Anogential distance; Vaginal opening; Estrus cycle length; Follicular development	**Genistein treatment: ** 50% reduction in chemically induced mammary tumorgenesis Increased mammary gland differentiation in immature rats leading to mammary gland less susceptible to mammary cancer No significant changes in fertility, number of male and female offspring, body weight, anogenital distance, vaginal opening, testes descent, estrus cycle, or follicular development among groups

#### 2.2.1. Reproductive Organ Morphology

Studies have shown that early exposure to soy isoflavone enhances differentiation of the mammary gland, leading to a mammary gland that is less susceptible to chemically-induced mammary cancer [37]. Furthermore, this effect is present at high levels of exposure, (subcutaneous injection of 500 mg genistein/kg body weight) and did not alter fertility and age at puberty onset. Genistein treatment resulted in fewer terminal end buds and advanced development and ductal elongation. It is known that terminal end buds are the most susceptible to carcinogens as they are the least mature terminal ductal structures [44]. A reduction in the numbers of terminal end buds can therefore explain the lower incidence of mammary cancer. Part of the terminal end bud differentiates according to each estrous cycle, giving rise to alveolar buds that consist of lobule structures that are more mature and less susceptible to chemical carcinogens [45]. Genistein treatment increased the number of lobules indicating a potential protective effect [45]. Previous findings have confirmed that early life exposure to estrogen causes differentiation in mammary tissue, leading to a mammary gland that is less susceptible to cancer [46]. The mechanism by which genistein influences mammary gland development is yet to be elucidated but these findings suggest genistein is exerting an estrogenic effect.

Another reproductive organ that is sensitive to isoflavone exposure is the uterus. Neonatal mice (PND 1–5) treated with genistein had greater uterine gland number (subcutaneous injection of 50 mg genistein/kg body weight) and increased uterine weight and epithelial cell height at higher doses (subcutaneous injection of 100 mg genistein/kg body weight) [28]. These results also suggest that genistein is mimicking the effect of estrogen on uterus, supporting the hypothesis that genistein acts like estrogen in the reproductive system [47]. Interestingly, daidzein did not cause such alterations in the uterus, suggesting that daidzein may not have a measurable estrogenic effect on the mouse uterus. In addition, a higher incidence of uterine adenocarcinoma, corpora lutea absence and oviduct abnormalities have been reported mice following treatment with genistein at subcutaneous injection of 50 mg genistein/kg body weight [35].

#### 2.2.2. Sexual Maturation and Endocrine Function

Estrogenic substances are known to alter endocrine function, especially when exposure happens during critical periods of development [48]. It was previously hypothesized that early exposure to compounds with estrogen-like activity may accelerate the age of puberty onset [49]. Earlier age at time of vaginal opening has been reported at doses of 40 mg genistein/kg body weight and this was administered from PND 1–21 combining both subcutaneous injection and oral gavage [21]. Interestingly however, at seemingly higher doses of genistein (subcutaneous injection of 100 mg genistein/kg body weight) no change in timing of puberty was observed [32]. Mice were only exposed from PND 1–5 however, compared to mice were exposed from PND 1–21, indicating that treatment duration may cause differing effects. Lower progesterone and differences in the amount of time spend in each phase of the estrous cycle without changes in estrous cycle length have also been observed after the 21 day treatment period [21]. Lordosis quotient may also be affected by genistein and daidzein exposure; however they have contrasting effects [35]. One study reported a low lordosis quotient in the genistein treated group, whereas a higher lordosis quotient was observed in the daidzein treated group [35]. These results suggest that genistein and daidzein may affect sexual differentiation of the brain, ultimately leading to differences in sexual behavior.

#### 2.2.3. Fertility

Impaired fertility in females has been documented after soy isoflavone exposure in the neonatal mouse model [31,33,35]. Neonatal genistein treatment resulted in pregnancy loss and was characterized by fewer implantation sites and increased resorption [31]. In the same study, increased numbers of corpora lutea after low dose genistein treatment (subcutaneous injection of 0.5 and 5 mg genistein/kg body weight) and reduced numbers of corpora lutea after higher doses (subcutaneous injection of 50 mg genistein/kg body weight) were observed. A prolonged estrous cycle without changes in serum estrogen, progesterone and testosterone before and during pregnancy was also reported at varying doses but showing a higher incidence of extended estrous at the highest subcutaneous dose of 50 mg genistein/kg body weight [31]. One study reported no corpora lutea after genistein treatment, smaller corpora lutea after daidzein treatment and both treatments resulted in smaller ovaries and these were at higher subcutaneous doses of approximately 83 mg genistein and daidzein/kg body weight [36]. Other abnormalities observed at subcutaneous doses of 50 mg genistein/kg body weight include the presence of multioocyte follicles (MOFs) [33]. Furthermore, MOFs were accompanied by prolonged nest breakdown and fewer oocytes undergoing apoptosis [33]. The implications of these findings are noteworthy since *in vitro* data suggest oocytes derived from MOFs have reduced fertilization capacity compared to single oocytes follicles [50]. More recently, however, Jefferson *et al.* has demonstrated that mice treated with the same subcutaneous dose of genistein had competent oocytes but these mice could not support normal implantation of control embryos and were unable to deliver live pups [35]. Using the same dose and route of administration, ER-α but not ER-β transcription was upregulated in mouse ovaries after exposure to genistein [29]. This is an important finding as the mechanism by which estrogen exerts its effect on the female reproductive tract is predominantly mediated through ER-α [51]. Early exposure to genistein compromises ovarian development and reproductive function in rodent models at serum levels that resemble those of human infants.

### 2.3. Male Reproductive Health: Animal Studies (Table 6)

#### 2.3.1. Reproductive Organ Differentiation and Morphology

Only two studies demonstrated a measurable effect of soy isoflavones on male reproductive development when exposure was limited to the suckling period (Table 6). In terms of organ morphology, seminiferous tubule lumen formation and a high sertoli cell nuclear volume that did not match the lumen volume per testis has been documented [38]. This measure is used to determine the capacity for pubertal spermatogenesis and thus indicates that spermatogenesis may be abnormal [38]. Interestingly, these effects were observed at relatively lower doses of genistein (subcutaneous injection of 4 mg genistein/kg body weight). In another report that administered genistein by oral gavage, no consistent morphological changes in the testes, epididymides, ventral prostate, and seminal vesicles were observed at oral doses of genistein up to 100 mg genistein/kg body weight [32]. 

**Table 6:** Studies in male animals examining the effects of soy isoflavone exposure during early life.

Objective	Sample Size	Subjects (age at time of intervention)	Intervention: Route of administration and dosage	Duration of Intervention	Reproductive Health Outcomes	Findings
To determine the effects of oral exposure to genistein on neonatal rats to assess human risk following oral ingestion of genistein [21]	Not determined	Alderley Park rats, PND 1	PND 1–6: SC Genistein: 0.2 or 2 mg genistein/kg body weight PND 7–21: Oral gavage Genistein: 4 mg/kg body weight 40 mg/kg body weight Control: corn oil	PND1–21	Serum FSH, LH, testosterone Preputial separation Testes descent	No consistent effects observed in males at either dose
To evaluate whether early exposure of neonates to genistein has any effect on the development of sexual organs and/or reproductive performance [32]	Not determined	Sprague-Dawley rats PND 1	Oral gavage Genistein: 12.5, 25, 50 or 100 mg/kg body weight Control: corn oil	PND 1–5	Preputial separation Fertility Sperm count Serum testosterone Histopathological changes of reproductive organs	Preputial separation, was not effected Male fertility was not effected Sperm counts and serum testosterone was not effected No histopathological changes in the gonads
		
To investigate whether neonatal exposure of estrogenic compounds altered pubertal spermatogenesis and whether the changes observed resulted in long‑term changes in testis size, mating or fertility [38]	Not determined	Wistar rats, PND 2	SC Genistein4 mg/kg body weight Control: corn oil	PND 2–18	Mating and fertility Sertoli cell and germ cell nuclear volume per testis Germ cell apoptotic index Seminiferous tubule lumen formation Plasma FSH	Few experienced impaired mating and fertility and low sample size was considered Slowed lumen formation Increased germ cell apoptotic rate High sertoli cell nuclear volume that did not match the lumen volume per testis Suppressed plasma FSH at PND 18
To establish if there are any biological consequences of consuming soy formula milk and to study the effects observed during and at the end of the feeding period which encompasses the period of the neonatal rise in testosterone in a non-human primate, the marmoset [39]	***n*** = 15/group (included 13 pairs of twins)	Marmoset monkeys 4–5 days old	Hand fed using 1 mL syringe(3–4 times on weekdays, 1–2 times on weekends) Cow’s milk formula Soy milk formula Formulas were prepared as per instructions and offered to the marmoset until feeding stopped Approximately 1.6–3.5 mg soy isoflavones/kg body weight	5–6 weeks	Histology: testes, epididymis, pituitary gland Sertoli and germ cell number per testes Leydig cell number Plasma testosterone	Soy formula fed males had mean testosterone levels were consistently lower than milk formula fed males No significant changes in numbers of sertoli cells or germ cells Leydig cell number increased by 74% Paired comparison in soy milk formula and cow’s milk formula co‑twins showed a 53–70% lower serum testosterone levels at day 35–45


To establish if there are any consequences of consuming soy formula milk and to study the effects observed on fertility and testicular structure in a non‑human primate, the marmoset [40]	***n*** = 7/group(14 total)	Marmoset co‑twin monkeys 4–5 days old	Hand fed using 1 mL syringe(3–4 times on weekdays, 1–2 times on weekends) Cow’s milk formula Soy milk formula Formulas were prepared as per instructions and offered to the marmoset until feeding stopped Approximately 1.6–3.5 mg soy isoflavones/kg body weight	5–6 weeks	Onset and progression of puberty based on testosterone levels Fertility Testicluar morphology	Normal progression of puberty Normal fertility Sertoli and leydig cell numbers/testes were significantly increased

#### 2.3.2. Male Sexual Maturation, Endocrine Function and Fertility

Most notable is the study that used twin marmoset monkeys [39] as it prompted many European countries to minimize the use of SBIF [52]. Importantly, unlike other studies, the marmoset monkeys were directly fed SBIF. In this study one twin was fed SBIF and the other was fed with cow’s milk formula beginning from day four or five of life. Of the twin pair, the marmoset fed SBIF, had a reduction in serum testosterone of 53–70% compared to its twin fed cow’s milk formula at 35–45 days of age [39]. Additionally, males fed cow’s milk formula had serum testosterone levels that are typical of the “neonatal testosterone rise” observed in human male neonates whereas the SBIF group had consistently reduced testosterone levels. At the end of the formula feeding an increased number of leydig cells were reported and may indicate compensation or adjustment for leydig failure. Of note is the fact that monkeys in this study were exposed to 1.6–3.5 mg isoflavones/kg body weight, which is less than half the level of exposure compared to a human infant consuming SBIF [14]. In a later study however, using the same subject group and feeding protocol normal fertility and progression of puberty was demonstrated [40]. Moreover, isoflavone metabolism in marmosets compared to human infants may be markedly different. A study in cynalogous monkeys demonstrated a markedly higher conversion of daidzein to equol, a more estrogenic isoflavone metabolite, than in human infants [49]. It is speculated that marmosets would also have a high rate of conversion from daidzein to equol.

There was no difference in testes weight in the preliminary results of a study examining SBIF consumption and reproductive health in neonatal pigs, which metabolize isoflavones in a similar way to human infants [52]. It should be noted however, that the sample size was relatively small, *n *= 4/group and these measurements were taken at postnatal day 21, prior to sexual maturity. Furthermore, normal testes weight does not provide confirmation for normal testicular development and multiple measures, as discussed in Table 2, should be used to determine if disruption in sexual maturation or endocrine function has occurred. 

Based on the data gathered from rodent studies, there appears to be no effect of soy isoflavones on sexual maturity in males. Preputial separation, fertility, sperm count and testosterone levels were unaffected by soy isoflavone treatment at oral doses of 100 mg genistein/kg body weight. Depressed plasma FSH has also been reported after genistein treatment (subcutaneous injection of 4 mg/kg body weight) [38] yet these results contrasted with those reported by others who observed no changes in FSH, LH, or testosterone after using a comparable dose of genistein when it was administered orally [21].

## 3. Conclusions and Future Directions

The biological effects of soy isoflavone exposure as a result of SBIF consumption are controversial and inconclusive. In summary, only one retrospective study has reported effects of feeding SBIF on health outcomes at adulthood and few studies have examined infant health after exposure to SBIF. While studies using a variety of animal models report negative effects of soy isoflavones exposure during development, it is unclear whether these data can be extrapolated to human infants. These studies do however suggest that further investigation of long term biological effects of early exposure to isoflavones is warranted. We feel that both studies in humans and using appropriate animal models are needed. Table 7 outlines various aspects of reproductive development that would be useful to measure in either humans or using animal models. Together, the findings from such studies will provide a more comprehensive understanding of the biological effects of isoflavones in SBIF on reproductive health.

**Table 7:** Future directions for human studies or using animal models.

Outcomes to Measure in Human Subjects	Outcomes to Measure in Animals
**Prospective Cohort** Sexual maturity Reproductive organ morphology, development and function Serum hormone levels Fertility Testicular, prostate, ovarian, uterine cancer Offspring characteristics (birth weight, sex ratio) **Retrospective Cohort** Serum hormone levels Fertility Reproductive organ morphology and function Testicular, prostate, ovarian, uterine cancer Offspring characteristics: (birth weight, sex ratio)	**Mechanism of Endocrine Disruption ** Hormone-specific effects on tissues Altered hormone receptor expression and/or activity Changes in gene expression Organ weight and histopathology Serum hormones at various life stages Transgenerational effects **Potential Outcomes Altered by Endocrine Disruption** Sexual maturity Fertility Testicular, prostate, ovarian, uterine cancer Offspring characteristics

### 3.1. Future Directions for Human Studies

Both retrospective and prospective studies are needed to determine how SBIF may be affecting human reproductive health. Prospective studies that monitor infants who are currently consuming SBIF, for abnormalities in reproductive organ development, such as the Beginnings Study [43], are needed. Prospective studies should focus not only on reproductive organ size, external genitalia and morphology, but also monitor for differences in pubertal onset and hormonal status. Compensated leydig cell failure/adjustment should also be measured in male infants. As demonstrated by [41] there are several techniques that are useful in order to physically examine the development of estrogen responsive tissues in infants. For example, breast bud diameter and vaginal cell specimens are useful outcomes to determine if estrogenization occurs [41]. It is suggested that vaginal cells be collected for >6 months and that specimens should be collected every one to two weeks for the first one to two months [41]. Vaginal bleeding and milk secretions, although rare, could also be documented. In males, the use of the urocytogram [53], which examines the hormonal responsiveness of urethral cells, could be assessed. Investigation to adult life is crucial since the impact of consuming SBIF may not be evident until adulthood. Although one retrospective study has been previously conducted [16] there is a need to increase the number of reproductive health outcomes measured. Performing ultrasounds, such as in the Beginnings Study, may be useful for identifying abnormalities in reproductive organs such as the occurrence of polycystic ovaries in women. Serum hormones in post-pubescent females should also be taken at specific time points in the menstrual cycle to obtain a quantifiable characterization of the menstrual cycle. This may more accurately reflect menstrual abnormalities, rather than using self reports alone. Sperm counts and sperm characteristics should also be measured in adult males in order to determine function of the male reproductive system. Fertility should be closely examined in this population as well since abnormalities in the reproductive system of both males and females do not necessarily compromise fertility. Because animal studies have shown a higher incidence of cancer in animals consuming high levels of soy isoflavones [34], cancer screening should be considered for adults who have consumed SBIF in infancy. As well, offspring of those who have consumed SBIF should be monitored for differences in growth and development. Many individuals in North America have consumed SBIF as infants, and so the potential to more extensively assess reproductive health is possible.

### 3.2. Future Directions for Animal Studies

Because sexual maturation is similar across species [54], animal models still provide a practical design for studying endocrine function. Numerous endocrine-mediated events involved in this progression in the rat for example, are comparable to other mammalian species such as humans [50]. In both humans [55] and rodents [56], pubertal onset is associated with similar physiological changes such as the attainment of a body mass, chronic inflammatory states, thyroid disease, and growth hormone deficiency. The control of gonadotropin releasing hormone (GnRH), the release of gonadotropins from the pituitary, and the steroid positive and negative feedback controls are fairly consistent across mammalian species [57]. Due to these species related similarities, the animal model may provide mechanistic support for investigations in humans. By demonstrating potential consequences of consuming soy isoflavones, such as increasing the number of MOFs or compromising embryo implantation, a better understanding of the potential impact of soy isoflavones is achieved. Additionally, by determining how soy isoflavones modulate gene expression and hormone receptor activity, potential biological effects may be predicted. There are various outcomes that cannot be measured in humans such as organ weight and histology. Such outcomes, which may be markers of higher rates of uterine cancer, primarily serve to guide the direction of soy isoflavone and SBIF research. Other environmental estrogens, such as diethlystillbesterol are known to cause harmful transgenerational effects [58,59] and there has been concern that soy isoflavones may act in a similar way. Animal models allow for controlled and time efficient transgenerational data to be collected. 

#### What would an ideal animal model be?

A mouse model could be refined to more closely characterize the human scenario. Ideally, the animal would be exposed orally, in order to ensure that first pass metabolism in the gut is occurring. As well, exposure should take place more than once per day, if technically possible, in order to mimic the multiple feedings that an infant would receive. Exposure should only take place during early postnatal development starting at the first day of life and during suckling, although the duration during suckling requires further study. Transgenerational studies, in order to characterize the offspring, are easily performed by breeding the treated animals to known controls and should also be conducted. Characterizing the ideal animal model for studying the effects of isoflavones in SBIF on human infants is an ongoing area of investigation.
